# Domestic Summary

**Published:** 1839-08-10

**Authors:** 


### DOMESTIC SUMMARY.

The milk sickness is a disease, of which no satisfactory account has been published. It is limited to certain portions of the United States, especially Kentucky, Ohio, Indiana, and Illinois. Indeed, we believe it is not only confined to those states, but to a very small portion of them. It occurs in a particular and often limited locality, and, after a few years, ceases. Cultivation seems to arrest it; whether this is always the case, is unknown.

The disease is supposed to be caused by poisonous vegetables eaten by cattle. When communicated to the human subject, the mouth or flesh of these animals produces the symptoms of most acrid poisons, and most evidently causes a local inflammation, with a depressing action upon the nervous system.

The following article is very incomplete; nevertheless, as the writer seems to have seen much of the disease, it may be interesting to our readers. We would invite our correspondents who reside in the districts of country where the disease prevails, to furnish us with a more satisfactory account of it. It would be interesting to the profession, both in the United States and in Europe.

*Extract from an Inaugural Thesis on Milk Sickness.* By David L. Simpson, M.D., of Owen County, Kentucky.—I have been raised in a section of country, where this disease has prevailed from its earliest settlement.

The general face of the country is broken, bu the soil is rich, producing lofty timber and a luxuriant crop of native vegetation, upon which cattle graze and fatten, and large numbers range, indiscriminately, upon hills and dales in the forests, and often escape the disease.

The cause of the disease is involved in obscurity, and it is only by analogy that we can arrive at any certain conclusion upon the subject; but, that a disease (sui generis) exists, or is produced *in* certain districts, which, *out* of those districts, was never known, is a fact, which the most sceptical can no longer deny.

I have often heard it observed, by the oldest settlers of the country, that excessively dry seasons were most productive of the disease, and my own observation goes to establish the position.

The spring and fall of the past year were both uncommonly dry, and I have never known a season so fatal to stock.

The following is a summary of facts, connected with the history of the disease.

1st. That it is confined to certain limits, and that the formation of the surface, soil, timber, &c., in those districts, corresponds with that of adjoining districts, where the disease was never known to prevail.

2d. That the milk of the cows produces the disease in calves, and persons that partake of it.

3d. That cows giving milk that is poisonous, are seldom affected by the disease themselves, while dry cattle that graze with them will sicken and die.

4th. That the disease is communicable to man through the medium of beef.

5th. That some districts, which in their native state were fatal to stock, but which are now in cultivation, are free from the disease.

It has been customary with the pioneers of the country, to belt or girdle their timber, several years before they intend to clear the land for cultivation, and the surface of such land is soon covered with rank vegetation. Many observers suppose that these *deadenings* (as they are called) are more dangerous to stock than the native forests; whence it has been inferred, that the disease is of vegetable origin, and that the noxious plant is eaten by our stock, only when other vegetation is generally scarce.

I have read a paper, written by G. W. Wright, of Cincinnati, Ohio, in which he advances the doctrine, that the disease is of *miasmatic* origin; but the fact of the locality of the disease, and that locality corresponding so nearly with adjoining healthy districts, is sufficient, without argument, to confute his theory.

He has advanced another idea, which is dangerously erroneous, viz.: that the disease cannot be communicated by beef, because a case was never known in Cincinnati, where beeves are constantly slaughtered that are driven from Kentucky, where the disease exists.

It is a fact well known to all acquainted with the disease in cattle, that those actually sick cannot be driven many miles, without exhibiting symptoms of disease; and if they are hurried, they will tremble, sicken, and die. Hence the great caution taken by the owners of stock, when they find them sick in the woodlands, to drive them very gently to their farms.

Many experiments have been made (with the milk of suspected cows) upon dogs, and the disease has been produced upon them in a few days. When cows die of the disease, great care is taken to keep valuable dogs from eating the flesh, because of its pernicious quality.

It is my opinion, that the cause may exist and lie dormant in the system, for a long time, and finally pass off without harm, unless it is developed by some exciting agent.

The prominent exciting causes of the disease in men, are over exercise, and excess in drinking *spirituous liquors*.

The symptoms of the disease, in its first stage, are, more or less languor, indisposition to move, and fatigue upon the slightest exertion.

The digestive functions are but slightly impaired, the appetite good, the tongue natural, and all the secretions seem undisturbed; but if the patient, in this condition, exercises so much as greatly to excite the circulation, or drinks freely of any stimulating liquor, that will have the same effect, the disease will be developed in its worst form, and be ushered in by nausea and vomiting of acrid bilious matter, a sensation of burning in the stomach, excessive thirst, obstinate constipation of the bowels, great depression of spirits, coldness of the extremities, offensive breath, a dull expression of the eye, great restlessness, a violent throbbing of the arteries of the abdomen, while the circulation in the extremities is below the natural standard, breathing laborious, coma, and at last death.

I have, though rarely, found my patients chilly, and when that symptom occurs, a slight febrile state of system follows, as a matter of course. Yet both are accidental attendants upon the disease. No pain is complained of, and the only cry of the patient is for cold water, ice, &c. &c. The sickness attendant on this disease is peculiar, and I do not recollect to have seen a patient, (that had all the symptoms fully developed) who was not under the impression that he would die of the attack. The pulse, in the early stage, is full and often slower than natural; but as the disease advances, it becomes quicker and smaller, and finally indistinct at the wrist, while the action of the heart and large arteries is violent. If I were to name a disease, to exemplify the term *congestive*, it would be this; as no disease that I have ever seen, is so completely of that character.

When I have found my patients with cold feet and hands, I have frequently asked them if they were not uncomfortably cool, and the reply has invariably been in the negative. If re-action ever ensues, I consider the patient out of danger.

The *treatment* of the disease is simple, and such as would naturally be suggested to an experienced physician.

I usually give alkalies, to correct the acidity of the stomach, and these are followed with cathartic medicine. Calomel, on account of its specific gravity, is preferable to any other cathartic, in the early stage of the disease. I have given it, in large doses, until seventy-five or one hundred grains were retained by the stomach, and afterwards, castor oil, followed with aloes, rhubarb, senna, and the like.

The drastic vegetable cathartics are seldom retained upon the stomach, though when the vomiting ceases before the bowels are acted upon, I often use them beneficially.

The motto of my preceptor is, "never to give up the ship," as success often attends persevering efforts, even after the hope of the patient and his friends is gone.

Early attention to the extremities is important. Frictions, with warm stimulating applications to the epigastrium and extremities, should never be neglected. I have often resorted to blisters over the stomach, but am not satisfied that they have any claim to superiority over sinapisms and other rubefacients.

Heated spirits of turpentine, applied to the surface, and covered with coarse brown paper, is a valuable remedy to restore the circulation. Enemas should never be neglected, and should be given every two or three hours, until the regular action of the bowels is restored.

A saturated solution of Glauber's salts is often used with success; but I have never confined myself to the use of any specific remedy. When one fails I try another, till I conquer the disease, or till the disease conquers my patient.

I have in a few cases tried croton oil, but cannot say much in favour of its use.

The great and only difficulty is to overcome the torpor of the bowels; and when that is accomplished, I feel satisfied. It is necessary to attend strictly to keeping the bowels soluble during convalescence, which can easily be effected with castor oil, or any simple cathartic. Flowers of sulphur, combined with cream of tartar, constitute a favourite remedy with me in this stage of the disease. The disease has often been treated successfully, by the early use of tartar emetic, and when it acts as a cathartic, is perhaps the best medicine. When it fails in this, the powers of the system often seem exhausted by the use of it, and hence I cannot regard it as a safe remedy.

Bleeding has been much urged by theorists,—but I have never tried it, and am induced to believe that it is unsafe, from the character of the disease, and from the effect that it is said to produce by those who have tried it.

My preceptor informed me, that in every case, when he tried the lancet, the patient seemed to sink immediately, and that he has never known a case of recovery, where bleeding was resorted to. On the other hand, I have found active stimulants beneficial, after the operation of purgatives, and frequently have given brandy, to relieve great prostration, even before medicine had acted upon the bowels.

I have now presented a simple detail of facts, as regards the history of the disease, its symptoms and treatment; and, for fear of wading into too deep water, I will try to get out, without venturing further into the intricacies of the subject.

*Transylvania Journal of Medicine.*

